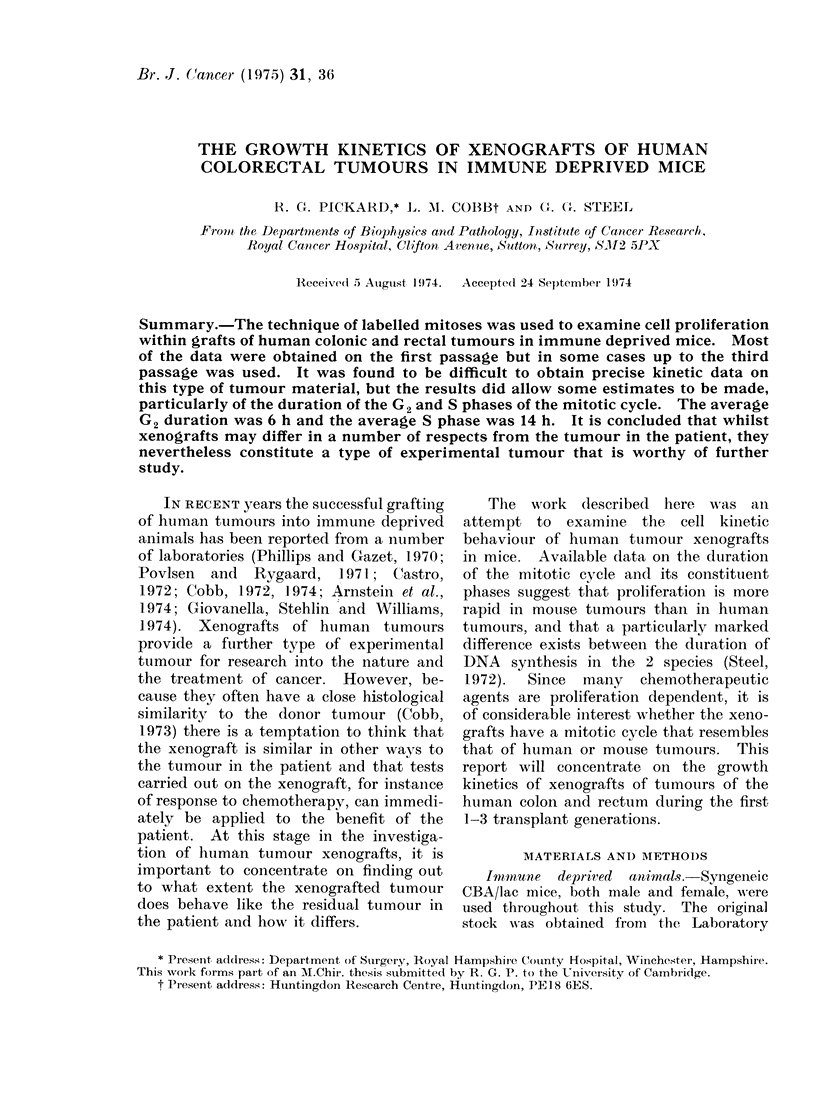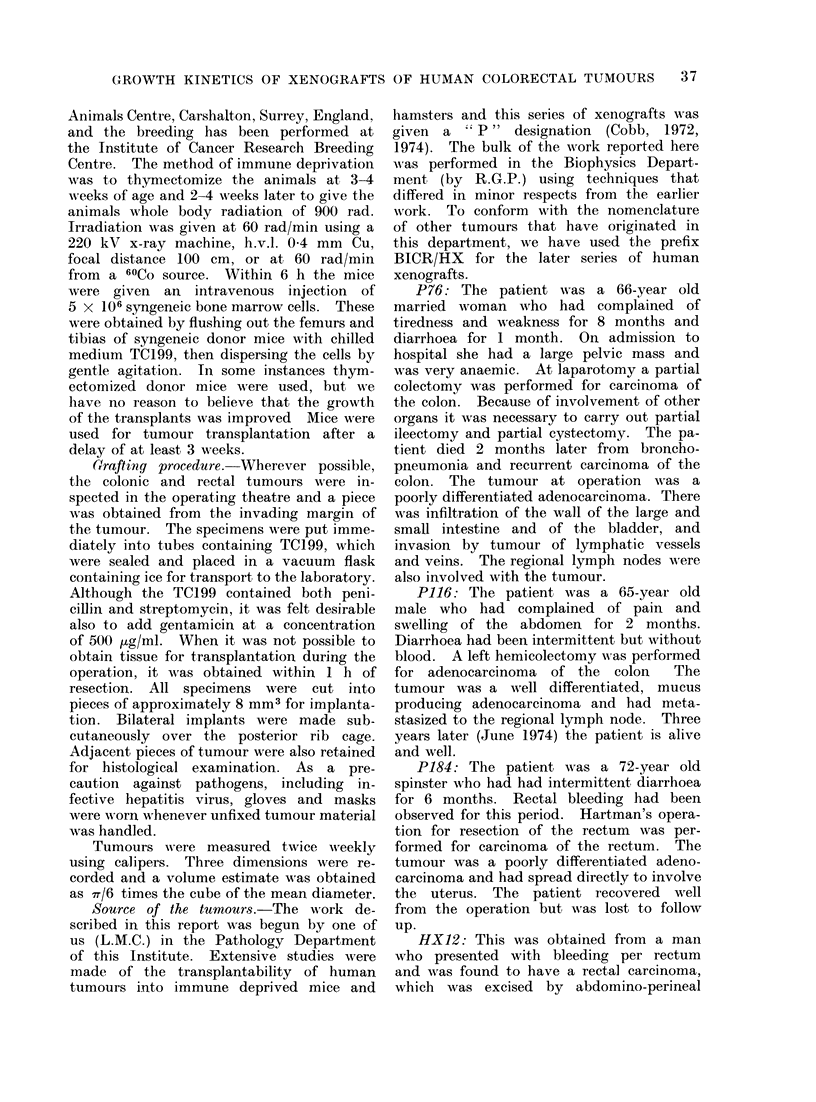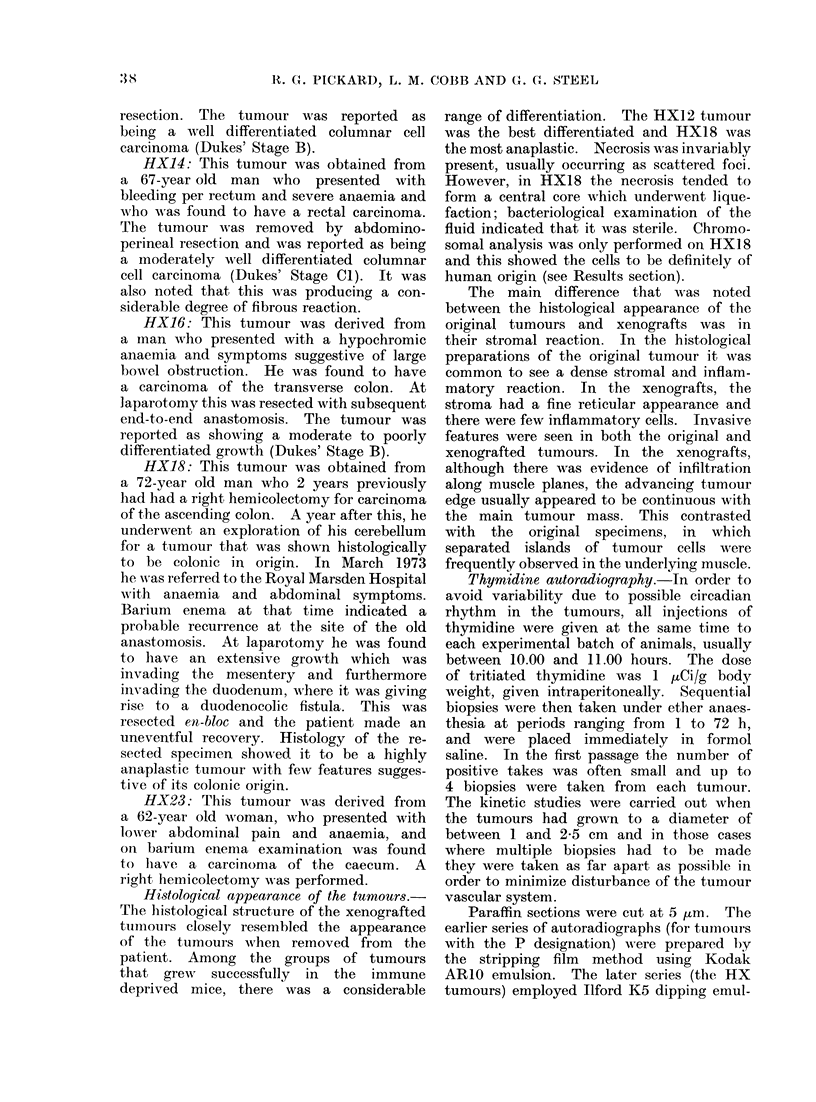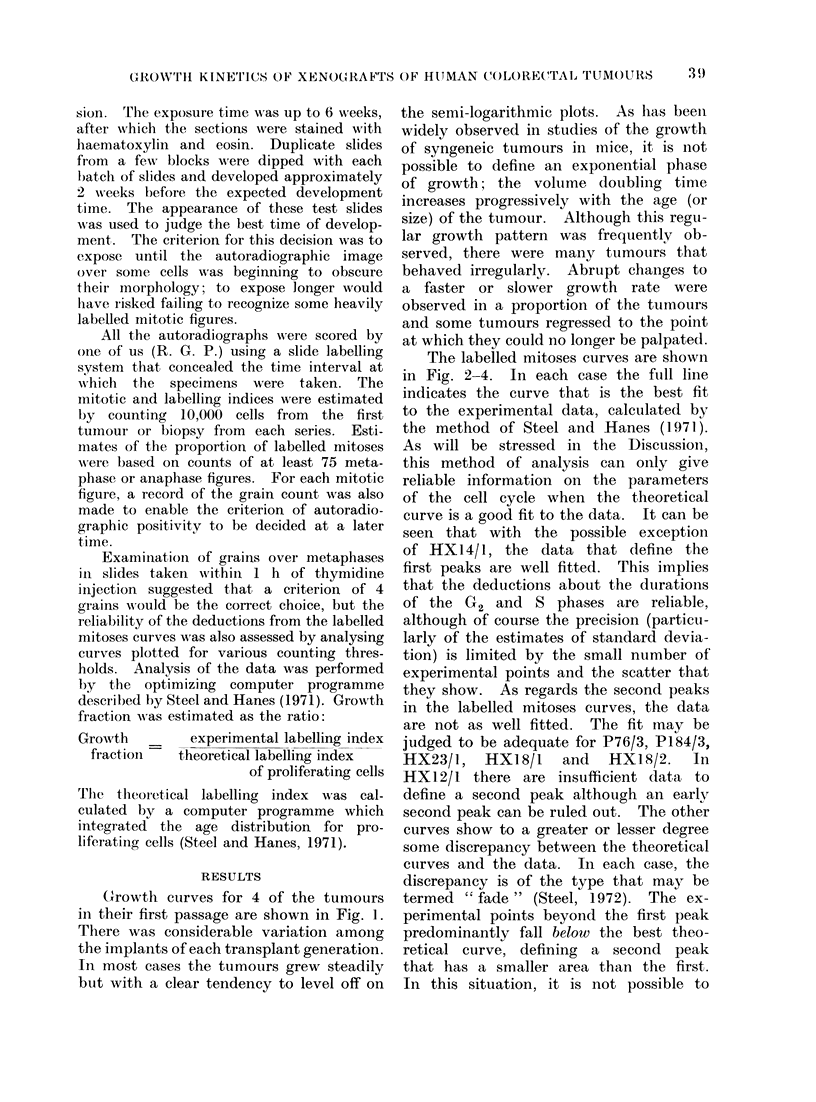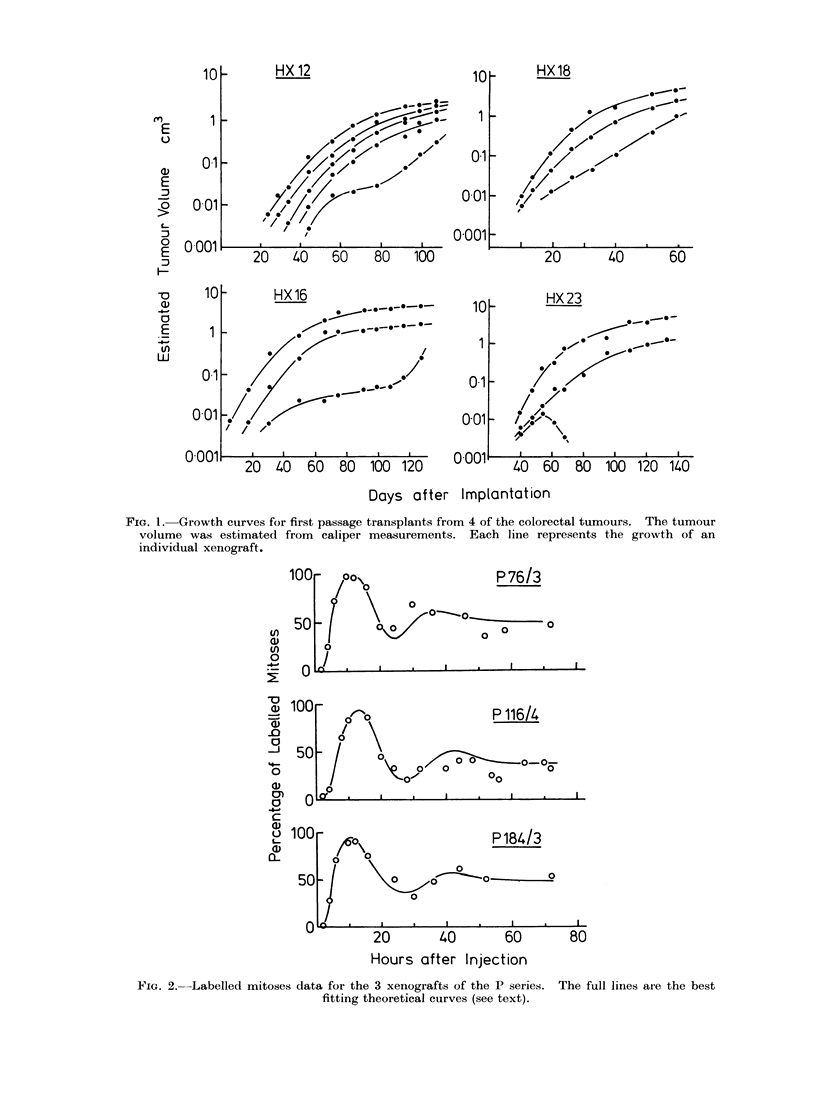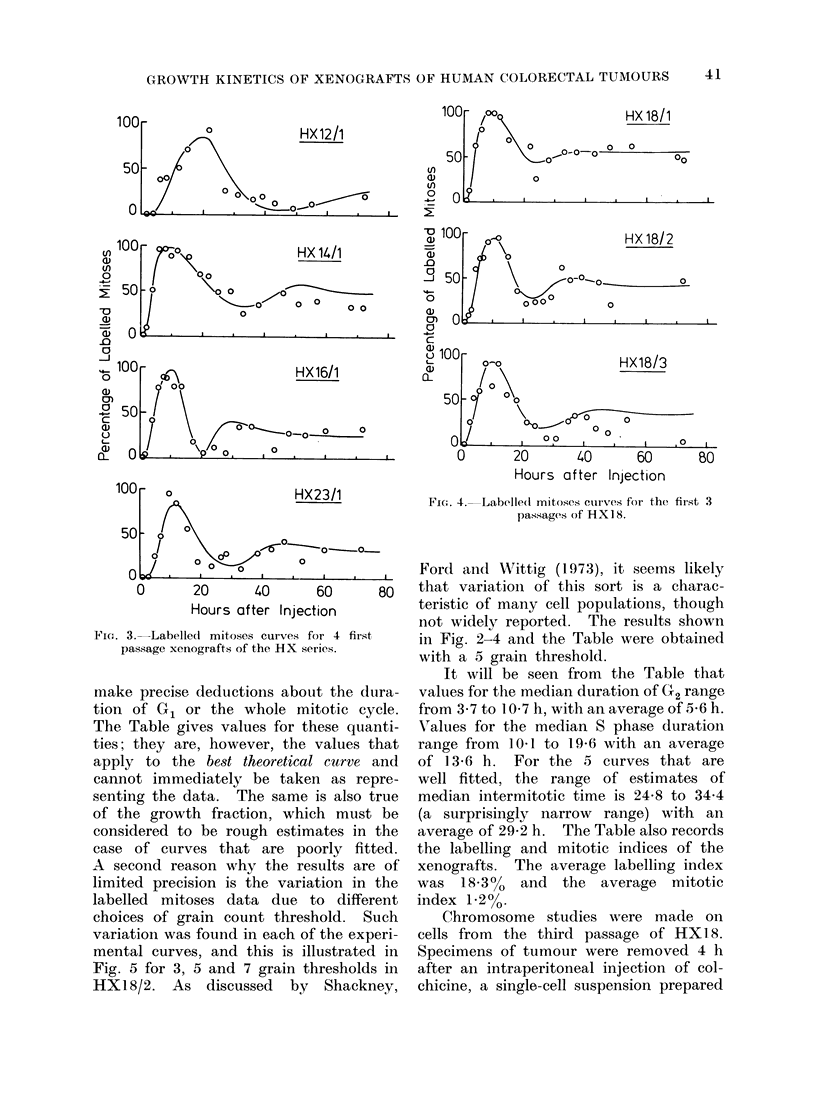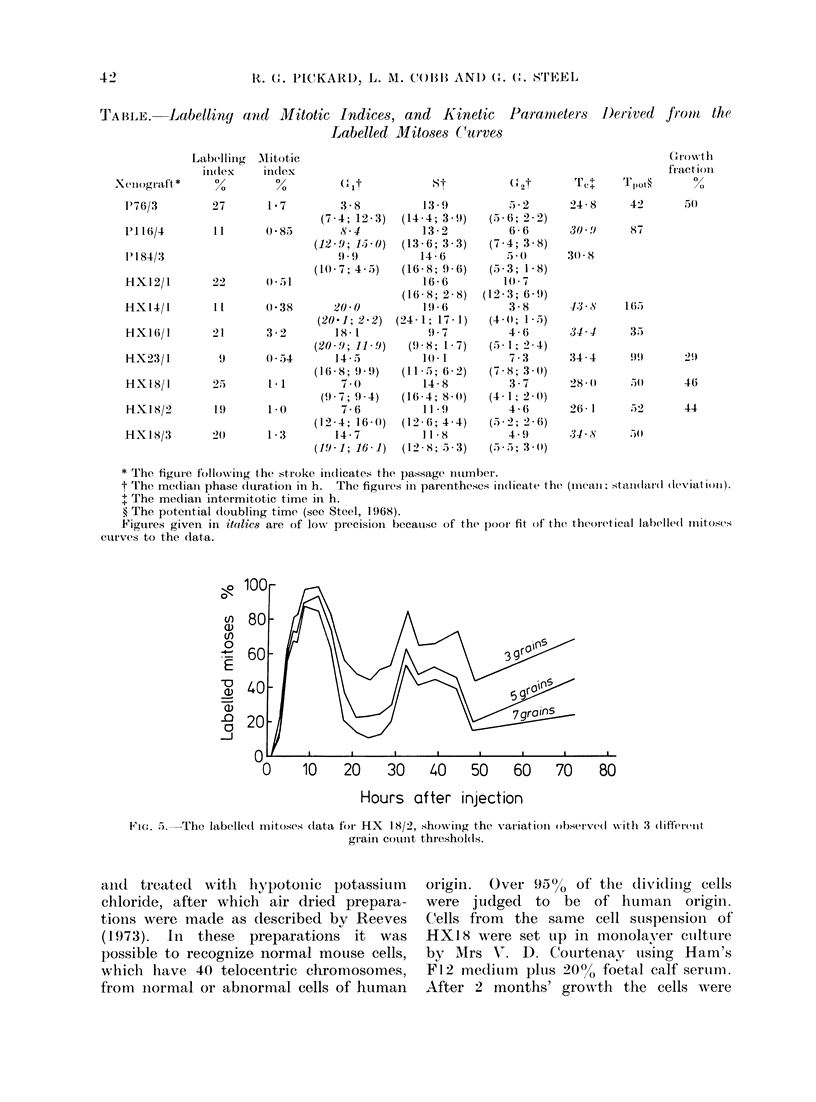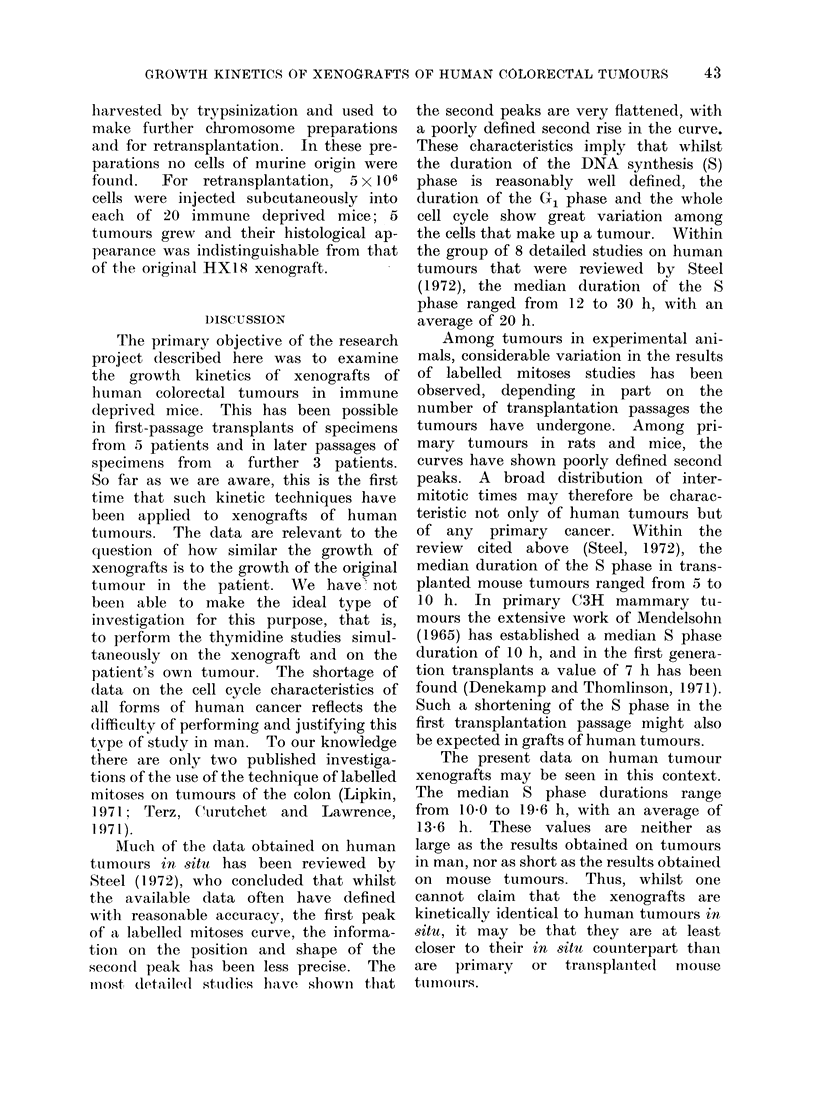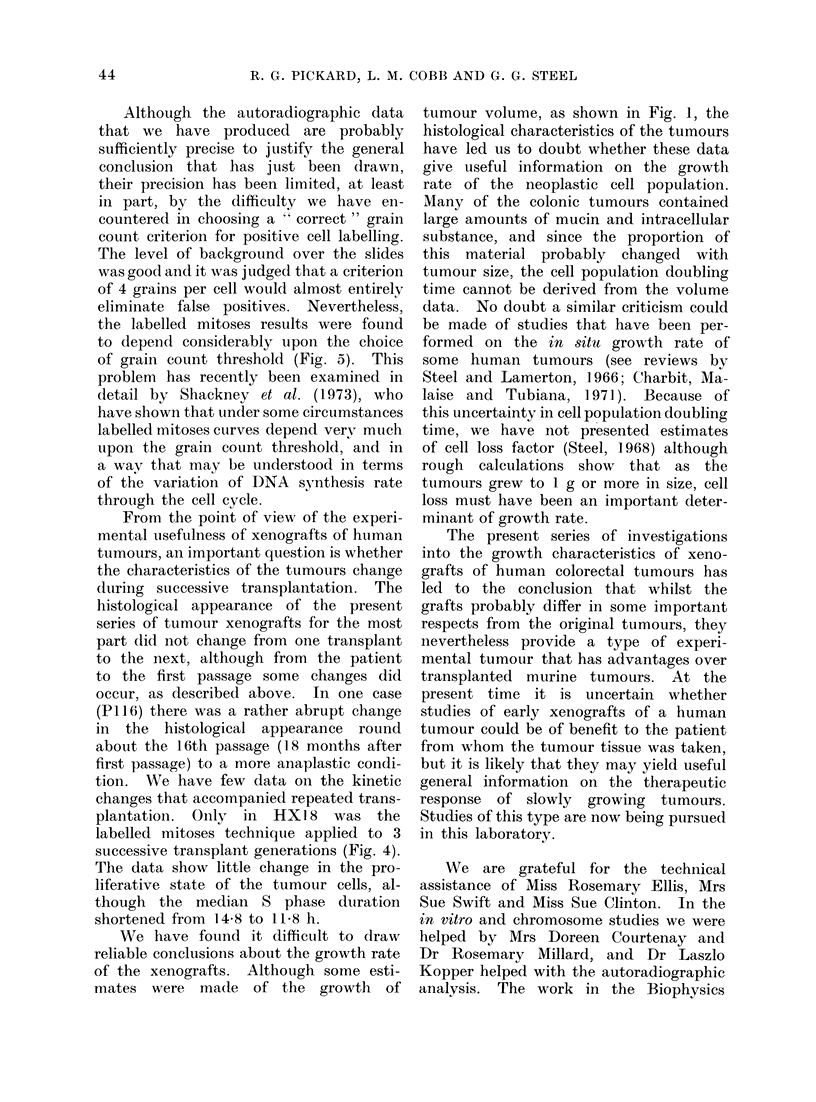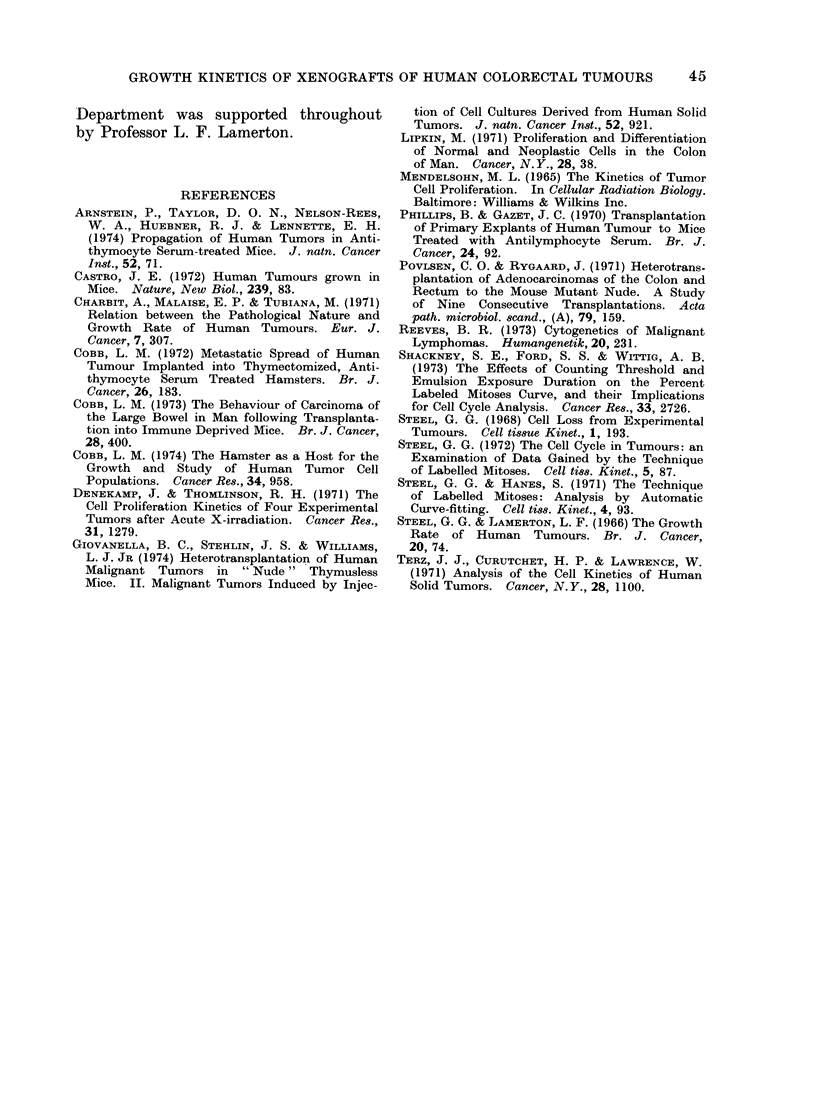# The growth kinetics of xenografts of human colorectal tumours in immune deprived mice.

**DOI:** 10.1038/bjc.1975.5

**Published:** 1975-01

**Authors:** R. G. Pickard, L. M. Cobb, G. G. Steel

## Abstract

The technique of labelled mitoses was used to examine cell proliferation within grafts of human colonic and rectal tumours in immune deprived mice. Most of the data were obtained on the first passage but in some cases up to the third passage was used. It was found to be difficult to obtain precise kinetic data on this type of tumour material, but the results did allow some estimates to be made, particularly of the duration of the G2 and S phases of the mitotic cycle. The average G2 duration was 6 h and the average S phase was 14 h. It is concluded that whilst xenografts may differ in a number of respects from the tumour in the patient, they nevertheless constitute a type of experimental tumour that is worthy of further study.


*Br. J. Cancer* (1975) **31**, 36

### THE GROWTH KINETICS OF XENOGRAFTS OF HUMAN COLORECTAL TUMOURS IN IMMUNE DEPRIVED MICE

R. G. PICKARD,* L. M. COBB† AND G. G. STEEL

*From the Departments of Biophysics and Pathology, Institute of Cancer Research, Royal Cancer Hospital, Clifton Avenue, Sutton, Surrey, SM2 5PX*

Received 5 August 1974. Accepted 24 September 1974

**Summary.—The technique of labelled mitoses was used to examine cell proliferation within grafts of human colonic and rectal tumours in immune deprived mice. Most of the data were obtained on the first passage but in some cases up to the third passage was used. It was found to be difficult to obtain precise kinetic data on this type of tumour material, but the results did allow some estimates to be made, particularly of the duration of the $G_2$ and S phases of the mitotic cycle. The average $G_2$ duration was 6 h and the average S phase was 14 h. It is concluded that whilst xenografts may differ in a number of respects from the tumour in the patient, they nevertheless constitute a type of experimental tumour that is worthy of further study.**

IN RECENT years the successful grafting of human tumours into immune deprived animals has been reported from a number of laboratories (Phillips and Gazet, 1970; Povlsen and Rygaard, 1971; Castro, 1972; Cobb, 1972, 1974; Arnstein *et al.*, 1974; Giovanella, Stehlin and Williams, 1974). Xenografts of human tumours provide a further type of experimental tumour for research into the nature and the treatment of cancer. However, because they often have a close histological similarity to the donor tumour (Cobb, 1973) there is a temptation to think that the xenograft is similar in other ways to the tumour in the patient and that tests carried out on the xenograft, for instance of response to chemotherapy, can immediately be applied to the benefit of the patient. At this stage in the investigation of human tumour xenografts, it is important to concentrate on finding out to what extent the xenografted tumour does behave like the residual tumour in the patient and how it differs.

The work described here was an attempt to examine the cell kinetic behaviour of human tumour xenografts in mice. Available data on the duration of the mitotic cycle and its constituent phases suggest that proliferation is more rapid in mouse tumours than in human tumours, and that a particularly marked difference exists between the duration of DNA synthesis in the 2 species (Steel, 1972). Since many chemotherapeutic agents are proliferation dependent, it is of considerable interest whether the xenografts have a mitotic cycle that resembles that of human or mouse tumours. This report will concentrate on the growth kinetics of xenografts of tumours of the human colon and rectum during the first 1–3 transplant generations.

### MATERIALS AND METHODS

*Immune deprived animals.*—Syngeneic CBA/lac mice, both male and female, were used throughout this study. The original stock was obtained from the Laboratory

* Present address: Department of Surgery, Royal Hampshire County Hospital, Winchester, Hampshire. This work forms part of an M.Chir. thesis submitted by R. G. P. to the University of Cambridge.

† Present address: Huntingdon Research Centre, Huntingdon, PE18 6ES.

Animals Centre, Carshalton, Surrey, England, and the breeding has been performed at the Institute of Cancer Research Breeding Centre. The method of immune deprivation was to thymectomize the animals at 3–4 weeks of age and 2–4 weeks later to give the animals whole body radiation of 900 rad. Irradiation was given at 60 rad/min using a 220 kV x-ray machine, h.v.l. 0·4 mm Cu, focal distance 100 cm, or at 60 rad/min from a $^{60}Co$ source. Within 6 h the mice were given an intravenous injection of $5 \times 10^6$ syngeneic bone marrow cells. These were obtained by flushing out the femurs and tibias of syngeneic donor mice with chilled medium TC199, then dispersing the cells by gentle agitation. In some instances thymectomized donor mice were used, but we have no reason to believe that the growth of the transplants was improved Mice were used for tumour transplantation after a delay of at least 3 weeks.

*Grafting procedure.*—Wherever possible, the colonic and rectal tumours were inspected in the operating theatre and a piece was obtained from the invading margin of the tumour. The specimens were put immediately into tubes containing TC199, which were sealed and placed in a vacuum flask containing ice for transport to the laboratory. Although the TC199 contained both penicillin and streptomycin, it was felt desirable also to add gentamicin at a concentration of 500 $\mu$g/ml. When it was not possible to obtain tissue for transplantation during the operation, it was obtained within 1 h of resection. All specimens were cut into pieces of approximately 8 $mm^3$ for implantation. Bilateral implants were made subcutaneously over the posterior rib cage. Adjacent pieces of tumour were also retained for histological examination. As a precaution against pathogens, including infective hepatitis virus, gloves and masks were worn whenever unfixed tumour material was handled.

Tumours were measured twice weekly using calipers. Three dimensions were recorded and a volume estimate was obtained as $\pi/6$ times the cube of the mean diameter.

*Source of the tumours.*—The work described in this report was begun by one of us (L.M.C.) in the Pathology Department of this Institute. Extensive studies were made of the transplantability of human tumours into immune deprived mice and hamsters and this series of xenografts was given a "P" designation (Cobb, 1972, 1974). The bulk of the work reported here was performed in the Biophysics Department (by R.G.P.) using techniques that differed in minor respects from the earlier work. To conform with the nomenclature of other tumours that have originated in this department, we have used the prefix BICR/HX for the later series of human xenografts.

*P76:* The patient was a 66-year old married woman who had complained of tiredness and weakness for 8 months and diarrhoea for 1 month. On admission to hospital she had a large pelvic mass and was very anaemic. At laparotomy a partial colectomy was performed for carcinoma of the colon. Because of involvement of other organs it was necessary to carry out partial ileectomy and partial cystectomy. The patient died 2 months later from bronchopneumonia and recurrent carcinoma of the colon. The tumour at operation was a poorly differentiated adenocarcinoma. There was infiltration of the wall of the large and small intestine and of the bladder, and invasion by tumour of lymphatic vessels and veins. The regional lymph nodes were also involved with the tumour.

*P116:* The patient was a 65-year old male who had complained of pain and swelling of the abdomen for 2 months. Diarrhoea had been intermittent but without blood. A left hemicolectomy was performed for adenocarcinoma of the colon The tumour was a well differentiated, mucus producing adenocarcinoma and had metastasized to the regional lymph node. Three years later (June 1974) the patient is alive and well.

*P184:* The patient was a 72-year old spinster who had had intermittent diarrhoea for 6 months. Rectal bleeding had been observed for this period. Hartman's operation for resection of the rectum was performed for carcinoma of the rectum. The tumour was a poorly differentiated adenocarcinoma and had spread directly to involve the uterus. The patient recovered well from the operation but was lost to follow up.

*HX12:* This was obtained from a man who presented with bleeding per rectum and was found to have a rectal carcinoma, which was excised by abdomino-perineal resection. The tumour was reported as being a well differentiated columnar cell carcinoma (Dukes' Stage B).

*HX14:* This tumour was obtained from a 67-year old man who presented with bleeding per rectum and severe anaemia and who was found to have a rectal carcinoma. The tumour was removed by abdomino-perineal resection and was reported as being a moderately well differentiated columnar cell carcinoma (Dukes' Stage C1). It was also noted that this was producing a considerable degree of fibrous reaction.

*HX16:* This tumour was derived from a man who presented with a hypochromic anaemia and symptoms suggestive of large bowel obstruction. He was found to have a carcinoma of the transverse colon. At laparotomy this was resected with subsequent end-to-end anastomosis. The tumour was reported as showing a moderate to poorly differentiated growth (Dukes' Stage B).

*HX18:* This tumour was obtained from a 72-year old man who 2 years previously had had a right hemicolectomy for carcinoma of the ascending colon. A year after this, he underwent an exploration of his cerebellum for a tumour that was shown histologically to be colonic in origin. In March 1973 he was referred to the Royal Marsden Hospital with anaemia and abdominal symptoms. Barium enema at that time indicated a probable recurrence at the site of the old anastomosis. At laparotomy he was found to have an extensive growth which was invading the mesentery and furthermore invading the duodenum, where it was giving rise to a duodenocolic fistula. This was resected *en-bloc* and the patient made an uneventful recovery. Histology of the resected specimen showed it to be a highly anaplastic tumour with few features suggestive of its colonic origin.

*HX23:* This tumour was derived from a 62-year old woman, who presented with lower abdominal pain and anaemia, and on barium enema examination was found to have a carcinoma of the caecum. A right hemicolectomy was performed.

*Histological appearance of the tumours.*—The histological structure of the xenografted tumours closely resembled the appearance of the tumours when removed from the patient. Among the groups of tumours that grew successfully in the immune deprived mice, there was a considerable range of differentiation. The HX12 tumour was the best differentiated and HX18 was the most anaplastic. Necrosis was invariably present, usually occurring as scattered foci. However, in HX18 the necrosis tended to form a central core which underwent liquefaction; bacteriological examination of the fluid indicated that it was sterile. Chromosomal analysis was only performed on HX18 and this showed the cells to be definitely of human origin (see Results section).

The main difference that was noted between the histological appearance of the original tumours and xenografts was in their stromal reaction. In the histological preparations of the original tumour it was common to see a dense stromal and inflammatory reaction. In the xenografts, the stroma had a fine reticular appearance and there were few inflammatory cells. Invasive features were seen in both the original and xenografted tumours. In the xenografts, although there was evidence of infiltration along muscle planes, the advancing tumour edge usually appeared to be continuous with the main tumour mass. This contrasted with the original specimens, in which separated islands of tumour cells were frequently observed in the underlying muscle.

*Thymidine autoradiography.*—In order to avoid variability due to possible circadian rhythm in the tumours, all injections of thymidine were given at the same time to each experimental batch of animals, usually between 10.00 and 11.00 hours. The dose of tritiated thymidine was 1 $\mu$Ci/g body weight, given intraperitoneally. Sequential biopsies were then taken under ether anaesthesia at periods ranging from 1 to 72 h, and were placed immediately in formol saline. In the first passage the number of positive takes was often small and up to 4 biopsies were taken from each tumour. The kinetic studies were carried out when the tumours had grown to a diameter of between 1 and 2·5 cm and in those cases where multiple biopsies had to be made they were taken as far apart as possible in order to minimize disturbance of the tumour vascular system.

Paraffin sections were cut at 5 $\mu$m. The earlier series of autoradiographs (for tumours with the P designation) were prepared by the stripping film method using Kodak AR10 emulsion. The later series (the HX tumours) employed Ilford K5 dipping emulsion. The exposure time was up to 6 weeks, after which the sections were stained with haematoxylin and eosin. Duplicate slides from a few blocks were dipped with each batch of slides and developed approximately 2 weeks before the expected development time. The appearance of these test slides was used to judge the best time of development. The criterion for this decision was to expose until the autoradiographic image over some cells was beginning to obscure their morphology; to expose longer would have risked failing to recognize some heavily labelled mitotic figures.

All the autoradiographs were scored by one of us (R. G. P.) using a slide labelling system that concealed the time interval at which the specimens were taken. The mitotic and labelling indices were estimated by counting 10,000 cells from the first tumour or biopsy from each series. Estimates of the proportion of labelled mitoses were based on counts of at least 75 metaphase or anaphase figures. For each mitotic figure, a record of the grain count was also made to enable the criterion of autoradiographic positivity to be decided at a later time.

Examination of grains over metaphases in slides taken within 1 h of thymidine injection suggested that a criterion of 4 grains would be the correct choice, but the reliability of the deductions from the labelled mitoses curves was also assessed by analysing curves plotted for various counting thresholds. Analysis of the data was performed by the optimizing computer programme described by Steel and Hanes (1971). Growth fraction was estimated as the ratio:

$$\text{Growth fraction} = \frac{\text{experimental labelling index}}{\text{theoretical labelling index of proliferating cells}}$$

The theoretical labelling index was calculated by a computer programme which integrated the age distribution for proliferating cells (Steel and Hanes, 1971).

### RESULTS

Growth curves for 4 of the tumours in their first passage are shown in Fig. 1. There was considerable variation among the implants of each transplant generation. In most cases the tumours grew steadily but with a clear tendency to level off on the semi-logarithmic plots. As has been widely observed in studies of the growth of syngeneic tumours in mice, it is not possible to define an exponential phase of growth; the volume doubling time increases progressively with the age (or size) of the tumour. Although this regular growth pattern was frequently observed, there were many tumours that behaved irregularly. Abrupt changes to a faster or slower growth rate were observed in a proportion of the tumours and some tumours regressed to the point at which they could no longer be palpated.

The labelled mitoses curves are shown in Fig. 2–4. In each case the full line indicates the curve that is the best fit to the experimental data, calculated by the method of Steel and Hanes (1971). As will be stressed in the Discussion, this method of analysis can only give reliable information on the parameters of the cell cycle when the theoretical curve is a good fit to the data. It can be seen that with the possible exception of HX14/1, the data that define the first peaks are well fitted. This implies that the deductions about the durations of the $G_2$ and S phases are reliable, although of course the precision (particularly of the estimates of standard deviation) is limited by the small number of experimental points and the scatter that they show. As regards the second peaks in the labelled mitoses curves, the data are not as well fitted. The fit may be judged to be adequate for P76/3, P184/3, HX23/1, HX18/1 and HX18/2. In HX12/1 there are insufficient data to define a second peak although an early second peak can be ruled out. The other curves show to a greater or lesser degree some discrepancy between the theoretical curves and the data. In each case, the discrepancy is of the type that may be termed "fade" (Steel, 1972). The experimental points beyond the first peak predominantly fall *below* the best theoretical curve, defining a second peak that has a smaller area than the first. In this situation, it is not possible to

FIG. 1.—Growth curves for first passage transplants from 4 of the colorectal tumours. The tumour volume was estimated from caliper measurements. Each line represents the growth of an individual xenograft.

FIG. 2.—Labelled mitoses data for the 3 xenografts of the P series. The full lines are the best fitting theoretical curves (see text).

FIG. 3.—Labelled mitoses curves for 4 first passage xenografts of the HX series.

FIG. 4.—Labelled mitoses curves for the first 3 passages of HX18.

make precise deductions about the duration of $G_1$ or the whole mitotic cycle. The Table gives values for these quantities; they are, however, the values that apply to the *best theoretical curve* and cannot immediately be taken as representing the data. The same is also true of the growth fraction, which must be considered to be rough estimates in the case of curves that are poorly fitted. A second reason why the results are of limited precision is the variation in the labelled mitoses data due to different choices of grain count threshold. Such variation was found in each of the experimental curves, and this is illustrated in Fig. 5 for 3, 5 and 7 grain thresholds in HX18/2. As discussed by Shackney, Ford and Wittig (1973), it seems likely that variation of this sort is a characteristic of many cell populations, though not widely reported. The results shown in Fig. 2–4 and the Table were obtained with a 5 grain threshold.

It will be seen from the Table that values for the median duration of $G_2$ range from 3·7 to 10·7 h, with an average of 5·6 h. Values for the median S phase duration range from 10·1 to 19·6 with an average of 13·6 h. For the 5 curves that are well fitted, the range of estimates of median intermitotic time is 24·8 to 34·4 (a surprisingly narrow range) with an average of 29·2 h. The Table also records the labelling and mitotic indices of the xenografts. The average labelling index was 18·3% and the average mitotic index 1·2%.

Chromosome studies were made on cells from the third passage of HX18. Specimens of tumour were removed 4 h after an intraperitoneal injection of colchicine, a single-cell suspension prepared

TABLE.—*Labelling and Mitotic Indices, and Kinetic Parameters Derived from the Labelled Mitoses Curves*

| Xenograft* | Labelling index % | Mitotic index % | $G_1$† | S† | $G_2$† | $T_c$‡ | $T_{pot}$§ | Growth fraction % |
|---|---|---|---|---|---|---|---|---|
| P76/3 | 27 | 1·7 | 3·8 (7·4; 12·3) | 13·9 (14·4; 3·9) | 5·2 (5·6; 2·2) | 24·8 | 42 | 50 |
| P116/4 | 11 | 0·85 | *8·4* (*12·9*; *15·0*) | 13·2 (13·6; 3·3) | 6·6 (7·4; 3·8) | *30·9* | 87 | |
| P184/3 | | | 9·9 (10·7; 4·5) | 14·6 (16·8; 9·6) | 5·0 (5·3; 1·8) | 30·8 | | |
| HX12/1 | 22 | 0·51 | | 16·6 (16·8; 2·8) | 10·7 (12·3; 6·9) | | | |
| HX14/1 | 11 | 0·38 | *20·0* (*20·1*; *2·2*) | 19·6 (24·1; 17·1) | 3·8 (4·0; 1·5) | *43·8* | 165 | |
| HX16/1 | 21 | 3·2 | 18·1 (*20·9*; *11·9*) | 9·7 (9·8; 1·7) | 4·6 (5·1; 2·4) | *34·4* | 35 | |
| HX23/1 | 9 | 0·54 | 14·5 (16·8; 9·9) | 10·1 (11·5; 6·2) | 7·3 (7·8; 3·0) | 34·4 | 99 | 29 |
| HX18/1 | 25 | 1·1 | 7·0 (9·7; 9·4) | 14·8 (16·4; 8·0) | 3·7 (4·1; 2·0) | 28·0 | 50 | 46 |
| HX18/2 | 19 | 1·0 | 7·6 (12·4; 16·0) | 11·9 (12·6; 4·4) | 4·6 (5·2; 2·6) | 26·1 | 52 | 44 |
| HX18/3 | 20 | 1·3 | 14·7 (*19·1*; *16·1*) | 11·8 (12·8; 5·3) | 4·9 (5·5; 3·0) | *34·8* | 50 | |

* The figure following the stroke indicates the passage number.

† The median phase duration in h. The figures in parentheses indicate the (mean; standard deviation).

‡ The median intermitotic time in h.

§ The potential doubling time (see Steel, 1968).

Figures given in *italics* are of low precision because of the poor fit of the theoretical labelled mitoses curves to the data.

FIG. 5.—The labelled mitoses data for HX 18/2, showing the variation observed with 3 different grain count thresholds.

and treated with hypotonic potassium chloride, after which air dried preparations were made as described by Reeves (1973). In these preparations it was possible to recognize normal mouse cells, which have 40 telocentric chromosomes, from normal or abnormal cells of human origin. Over 95% of the dividing cells were judged to be of human origin. Cells from the same cell suspension of HX18 were set up in monolayer culture by Mrs V. D. Courtenay using Ham's F12 medium plus 20% foetal calf serum. After 2 months' growth the cells were harvested by trypsinization and used to make further chromosome preparations and for retransplantation. In these preparations no cells of murine origin were found. For retransplantation, $5 \times 10^6$ cells were injected subcutaneously into each of 20 immune deprived mice; 5 tumours grew and their histological appearance was indistinguishable from that of the original HX18 xenograft.

### DISCUSSION

The primary objective of the research project described here was to examine the growth kinetics of xenografts of human colorectal tumours in immune deprived mice. This has been possible in first-passage transplants of specimens from 5 patients and in later passages of specimens from a further 3 patients. So far as we are aware, this is the first time that such kinetic techniques have been applied to xenografts of human tumours. The data are relevant to the question of how similar the growth of xenografts is to the growth of the original tumour in the patient. We have not been able to make the ideal type of investigation for this purpose, that is, to perform the thymidine studies simultaneously on the xenograft and on the patient's own tumour. The shortage of data on the cell cycle characteristics of all forms of human cancer reflects the difficulty of performing and justifying this type of study in man. To our knowledge there are only two published investigations of the use of the technique of labelled mitoses on tumours of the colon (Lipkin, 1971; Terz, Curutchet and Lawrence, 1971).

Much of the data obtained on human tumours *in situ* has been reviewed by Steel (1972), who concluded that whilst the available data often have defined with reasonable accuracy, the first peak of a labelled mitoses curve, the information on the position and shape of the second peak has been less precise. The most detailed studies have shown that the second peaks are very flattened, with a poorly defined second rise in the curve. These characteristics imply that whilst the duration of the DNA synthesis (S) phase is reasonably well defined, the duration of the $G_1$ phase and the whole cell cycle show great variation among the cells that make up a tumour. Within the group of 8 detailed studies on human tumours that were reviewed by Steel (1972), the median duration of the S phase ranged from 12 to 30 h, with an average of 20 h.

Among tumours in experimental animals, considerable variation in the results of labelled mitoses studies has been observed, depending in part on the number of transplantation passages the tumours have undergone. Among primary tumours in rats and mice, the curves have shown poorly defined second peaks. A broad distribution of intermitotic times may therefore be characteristic not only of human tumours but of any primary cancer. Within the review cited above (Steel, 1972), the median duration of the S phase in transplanted mouse tumours ranged from 5 to 10 h. In primary C3H mammary tumours the extensive work of Mendelsohn (1965) has established a median S phase duration of 10 h, and in the first generation transplants a value of 7 h has been found (Denekamp and Thomlinson, 1971). Such a shortening of the S phase in the first transplantation passage might also be expected in grafts of human tumours.

The present data on human tumour xenografts may be seen in this context. The median S phase durations range from 10·0 to 19·6 h, with an average of 13·6 h. These values are neither as large as the results obtained on tumours in man, nor as short as the results obtained on mouse tumours. Thus, whilst one cannot claim that the xenografts are kinetically identical to human tumours *in situ*, it may be that they are at least closer to their *in situ* counterpart than are primary or transplanted mouse tumours.

Although the autoradiographic data that we have produced are probably sufficiently precise to justify the general conclusion that has just been drawn, their precision has been limited, at least in part, by the difficulty we have encountered in choosing a " correct " grain count criterion for positive cell labelling. The level of background over the slides was good and it was judged that a criterion of 4 grains per cell would almost entirely eliminate false positives. Nevertheless, the labelled mitoses results were found to depend considerably upon the choice of grain count threshold (Fig. 5). This problem has recently been examined in detail by Shackney *et al.* (1973), who have shown that under some circumstances labelled mitoses curves depend very much upon the grain count threshold, and in a way that may be understood in terms of the variation of DNA synthesis rate through the cell cycle.

From the point of view of the experimental usefulness of xenografts of human tumours, an important question is whether the characteristics of the tumours change during successive transplantation. The histological appearance of the present series of tumour xenografts for the most part did not change from one transplant to the next, although from the patient to the first passage some changes did occur, as described above. In one case (P116) there was a rather abrupt change in the histological appearance round about the 16th passage (18 months after first passage) to a more anaplastic condition. We have few data on the kinetic changes that accompanied repeated transplantation. Only in HX18 was the labelled mitoses technique applied to 3 successive transplant generations (Fig. 4). The data show little change in the proliferative state of the tumour cells, although the median S phase duration shortened from 14·8 to 11·8 h.

We have found it difficult to draw reliable conclusions about the growth rate of the xenografts. Although some estimates were made of the growth of tumour volume, as shown in Fig. 1, the histological characteristics of the tumours have led us to doubt whether these data give useful information on the growth rate of the neoplastic cell population. Many of the colonic tumours contained large amounts of mucin and intracellular substance, and since the proportion of this material probably changed with tumour size, the cell population doubling time cannot be derived from the volume data. No doubt a similar criticism could be made of studies that have been performed on the *in situ* growth rate of some human tumours (see reviews by Steel and Lamerton, 1966; Charbit, Malaise and Tubiana, 1971). Because of this uncertainty in cell population doubling time, we have not presented estimates of cell loss factor (Steel, 1968) although rough calculations show that as the tumours grew to 1 g or more in size, cell loss must have been an important determinant of growth rate.

The present series of investigations into the growth characteristics of xenografts of human colorectal tumours has led to the conclusion that whilst the grafts probably differ in some important respects from the original tumours, they nevertheless provide a type of experimental tumour that has advantages over transplanted murine tumours. At the present time it is uncertain whether studies of early xenografts of a human tumour could be of benefit to the patient from whom the tumour tissue was taken, but it is likely that they may yield useful general information on the therapeutic response of slowly growing tumours. Studies of this type are now being pursued in this laboratory.

We are grateful for the technical assistance of Miss Rosemary Ellis, Mrs Sue Swift and Miss Sue Clinton. In the *in vitro* and chromosome studies we were helped by Mrs Doreen Courtenay and Dr Rosemary Millard, and Dr Laszlo Kopper helped with the autoradiographic analysis. The work in the Biophysics

Department was supported throughout by Professor L. F. Lamerton.

### REFERENCES

ARNSTEIN, P., TAYLOR, D. O. N., NELSON-REES, W. A., HUEBNER, R. J. & LENNETTE, E. H. (1974) Propagation of Human Tumors in Antithymocyte Serum-treated Mice. *J. natn. Cancer Inst.*, **52**, 71.

CASTRO, J. E. (1972) Human Tumours grown in Mice. *Nature, New Biol.*, **239**, 83.

CHARBIT, A., MALAISE, E. P. & TUBIANA, M. (1971) Relation between the Pathological Nature and Growth Rate of Human Tumours. *Eur. J. Cancer*, **7**, 307.

COBB, L. M. (1972) Metastatic Spread of Human Tumour Implanted into Thymectomized, Antithymocyte Serum Treated Hamsters. *Br. J. Cancer*, **26**, 183.

COBB, L. M. (1973) The Behaviour of Carcinoma of the Large Bowel in Man following Transplantation into Immune Deprived Mice. *Br. J. Cancer*, **28**, 400.

COBB, L. M. (1974) The Hamster as a Host for the Growth and Study of Human Tumor Cell Populations. *Cancer Res.*, **34**, 958.

DENEKAMP, J. & THOMLINSON, R. H. (1971) The Cell Proliferation Kinetics of Four Experimental Tumors after Acute X-irradiation. *Cancer Res.*, **31**, 1279.

GIOVANELLA, B. C., STEHLIN, J. S. & WILLIAMS, L. J. JR (1974) Heterotransplantation of Human Malignant Tumors in "Nude" Thymusless Mice. II. Malignant Tumors Induced by Injection of Cell Cultures Derived from Human Solid Tumors. *J. natn. Cancer Inst.*, **52**, 921.

LIPKIN, M. (1971) Proliferation and Differentiation of Normal and Neoplastic Cells in the Colon of Man. *Cancer, N.Y.*, **28**, 38.

MENDELSOHN, M. L. (1965) The Kinetics of Tumor Cell Proliferation. In *Cellular Radiation Biology*. Baltimore: Williams & Wilkins Inc.

PHILLIPS, B. & GAZET, J. C. (1970) Transplantation of Primary Explants of Human Tumour to Mice Treated with Antilymphocyte Serum. *Br. J. Cancer*, **24**, 92.

POVLSEN, C. O. & RYGAARD, J. (1971) Heterotransplantation of Adenocarcinomas of the Colon and Rectum to the Mouse Mutant Nude. A Study of Nine Consecutive Transplantations. *Acta path. microbiol. scand.*, (A), **79**, 159.

REEVES, B. R. (1973) Cytogenetics of Malignant Lymphomas. *Humangenetik*, **20**, 231.

SHACKNEY, S. E., FORD, S. S. & WITTIG, A. B. (1973) The Effects of Counting Threshold and Emulsion Exposure Duration on the Percent Labeled Mitoses Curve, and their Implications for Cell Cycle Analysis. *Cancer Res.*, **33**, 2726.

STEEL, G. G. (1968) Cell Loss from Experimental Tumours. *Cell tissue Kinet.*, **1**, 193.

STEEL, G. G. (1972) The Cell Cycle in Tumours: an Examination of Data Gained by the Technique of Labelled Mitoses. *Cell tiss. Kinet.*, **5**, 87.

STEEL, G. G. & HANES, S. (1971) The Technique of Labelled Mitoses: Analysis by Automatic Curve-fitting. *Cell tiss. Kinet.*, **4**, 93.

STEEL, G. G. & LAMERTON, L. F. (1966) The Growth Rate of Human Tumours. *Br. J. Cancer*, **20**, 74.

TERZ, J. J., CURUTCHET, H. P. & LAWRENCE, W. (1971) Analysis of the Cell Kinetics of Human Solid Tumors. *Cancer, N.Y.*, **28**, 1100.